# Multi-target Mechanisms of Si-Ni-San on Anxious Insomnia: An Example of Network-pharmacology and Molecular Docking Analysis

**DOI:** 10.2174/0109298673299665240924090617

**Published:** 2024-10-09

**Authors:** Chih Ting Lin, Hsin Yi Lin, Wen Huang Peng, Lung Yuan Wu

**Affiliations:** 1 The School of Chinese Medicine for Post-Baccalaureate, I-Shou University, No. 8, Yida Rd., Jiaosu Village Yanchao District, Kaohsiung City, 82445, Taiwan;; 2 Department of Chinese Medicine, E Da Cancer Hospital, I Shou University, No. 21, Yida Rd., Jiaosu Village Yanchao District, Kaohsiung City, 82445, Taiwan;; 3 School of Chinese Pharmaceutical Sciences and Chinese Medicine Resources, China Medical University, No.91, Hsueh-Shih Road, Taichung, 40402, Taiwan;; 4 Wu Lung-Yuan Chinese Medicine Clinic, 3 F, No. 131, Section 1, Roosevelt Rd., Zhongzheng District, Taipei City, 10093, Taiwan;; 5 Graduate Institute of Chinese Pharmaceutical Sciences, College of Chinese Medicine, China Medical University, No.91, Hsueh-Shih Road, Taichung, 40421, Taiwan

**Keywords:** Si-Ni-San, anxious insomnia, network pharmacology, molecular docking, gene oncology, IL6

## Abstract

**Background and objective:**

Based on comprehensive network-pharmacology and molecular docking analysis, this study was intended to unveil the multiple mechanisms of Si-Ni-San (SNS) in treating anxious insomnia.

**Methods:**

The compounds of SNS were meticulously analyzed, selected and standardized with references to their pharmacological attributes. The components included chaihu (*Bupleurum chinense DC.*), baishao (*Paeonia lactiflora Pall.*), zhishi (*Citrus aurantium L.*) and gancao (*Glycyrrhiza uralensis Fisch. ex DC.*). We used the Traditional Chinese Medicine System Pharmacology (TCMSP) Database, Traditional Chinese Medicines Integrated Database (TCMID), GeneCards database, therapeutic target database (TTD) and comparative toxicogenomic database (CTD) to construct the components-compounds-targets networks and used Cytoscape 3.9.1 software to visualize the outcome. Afterwards, the STRING database and Cytoscape 3.9.1 software were utilized to construct and visualize the protein-protein interaction (PPI) network analysis. In addition, the Gene Ontology (GO) and Kyoto Encyclopedia of Genes and Genomes (KEGG) pathway enrichment analysis were also conducted through the Database for Annotation, Visualization, and Integrated Discovery (DAVID). The molecular docking program was carried out using AutoDock 4.2 software to understand interactions between target receptors and compound ligands selected for study.

**Results:**

We thoroughly sorted and filtered 31 pharmacologically active compounds from SNS. Subsequently, several potential target genes were predicted, of which there were 59 target genes distinctly associated with anxious insomnia. The PPI analysis indicated that the core target proteins included AKT1, IL6, TNF, SLC6A4, MAOA and GABRA2. The results of our study indicated that SNS potentially remediates anxious insomnia by reducing inflammation, neurodegeneration, and cell apoptosis of neurons. In addition, GO and KEGG enrichment analysis results indicated that SNS could modulate multiple aspects of anxious insomnia through mechanisms related to pathways of neuroactive ligand-receptor interaction. These pathways include various kinds of synaptic transmission pathways, and anti-inflammatory activity associated with response pathways. When we compared the components-compounds-targets networks and the compounds-targets-synaptic pathways networks, the five active compounds, including beta-Sitosterol, Kaempferol, Tetramethoxyluteolin, Isorhamnetin and Shinpterocarpin, were selected to conduct molecular docking experiments. Eleven target proteins, (AKT1, SLC6A4, ADRB2, MAOA, ACHE, ESR1, CYP3A4, CHRNA7, GABRA2, HTR2A and NOS3), which also play significant roles in regulating serotonergic, cholinergic, dopaminergic and GABAergic systems in the PPI network, were selected to act as receptors in molecular docking trials. The results showed that docking pairs isorhamnetin-AKT1, isorhamnetin-SLC6A4, β-sitosterol-MAOA, β-sitosterol-ACHE, isorhamnetin-CHRNA7 and shinpterocarpin-GABRA2 provided the most stable conformations of ligand-receptor binding between key compounds and core target proteins in the SNS.

**Conclusion:**

In the study, we offer a computational result, revealing that SNS may alleviate sleep disorders associated with anxiety through a “multi-compounds, multi-targets, and multi-pathways” mechanism. The network-pharmacology and molecular docking outcomes could theoretically confirm the anti-anxiety and anti-insomnia effects of SNS. Although this research is purely statistical and systematic without empirical validation, it serves as a stepping stone and cornerstone for subsequent experimental investigations.

## 1 INTRODUCTION

Those who suffer from generalized anxiety disorder may experience feeling unfocused, suffer disquiet, or anxiety for no obvious reason. These anxiety symptoms may have specific aggravating factors. It was reported that the combined lifetime prevalence of generalized anxiety disorder was 3.7% [1]. The manifestations of generalized anxiety disorder also had comorbid conditions with other personality disorders, such as insomnia [2].

Insomnia, a common phenomenon in approximately 10% of the adult population in Taiwan, causes substantial impairments in a patient’s quality of life and social interactions. The primary symptom of insomnia is poor quality or insufficient quantity of sleep, including being unable to fall asleep (14.6%) or to stay asleep (13.4%) [3]. Daytime symptoms for insomnia patients include fatigue, low energy, cognitive impairment, and emotional disturbances [4]. According to ICSD-3/DSM-5^TM^ diagnostic criteria, a significant proportion of patients with insomnia experienced comorbid anxiety [4-6]. Additionally, in patients with comorbid generalized anxiety disorder and insomnia, neurologically, there was significant network segregation and increased hyperarousal of the posterior cingulate cortex [7, 8]. Similar clinical treatments can be used for generalized anxiety disorder and insomnia. These treatments include cognitive behavioral therapy, selective serotonin reuptake inhibitors (SSRI) or benzodiazepine (BZD) [9]. Cognitive behavioral therapy is designed to change sleep-related behaviors. SSRIs have been widely prescribed to increase the level of serotonin in the brain. BZDs were approved to treat insomnia, generalized anxiety disorder, and social anxiety disorder. However, therapy or medications may be problematic or have negative impacts, such as the lack of availability or time commitment required for cognitive behavioral therapy and a multitude of reported side effects of SSRIs and BZDs [10-12]. It is possible that traditional Chinese medicines (TCMs) or medicinal herbs could potentially offer better alternative approaches with fewer side effects [13].

Si-Ni-San (SNS), a famous Chinese herbal formula, has traditionally been utilized to harmonize the functions of the liver and spleen [14]. SNS is composed of four botanical drugs in a ratio of 1:1:1:1. The plant based origin of these drugs include chaihu *(Bupleurum chinense* DC.), baishao (*Paeonia lactiflora* Pall.), zhishi (*Citrus aurantium* L.) and gancao (*Glycyrrhiza uralensis* Fisch.) [15]. SNS has been clinically studied for the improvement of mental diseases, including chronic mental illness and psychiatric disorders [16]. For example, SNS imparts sedative functions by prolonging slow-wave sleep and rapid eye movement sleep [17]. Clinical trials have confirmed that SNS is effective in alleviating insomnia, especially anxiety-induced insomnia. After receiving SNS treatment, patients displayed significant score decreases in their sleep measurement scores (Pittsburgh Sleep Quality Index and Sleep Disorder Rating Scale) as well as in their anxiety assessment scores (Self-Rating Anxiety Scale and Hamilton Anxiety Rating Scale) [18, 19]. Paeoniflorin in SNS simultaneously exerts anti-depressive and prokinetic effects on rats subjected to forced swimming tests [20]. Moreover, clinical trials have demonstrated that in comparison to SSRI and BZD, SNS therapy notably presents a reduced spectrum of adverse effects, encompassing symptoms like nausea, vomiting, daytime sleepiness, dizziness, headaches, and elevated blood pressure [21, 22]. Therefore, SNS could offer an alternative treatment that is effective but incurs relatively fewer side-effects for anxious insomnia.

Network pharmacology and molecular docking analysis research shows that SNS helps suppress the activation of dopaminergic synapses and amphetamine addiction signaling pathways [23]. Mechanisms by which SNS could act against anxious insomnia (the comorbidity of generalized anxiety disorder and insomnia) have yet to be clarified, in part because of the complex ingredients in SNS. In this study, we employed a systematic, computational, biology-based approach and molecular biology simulation technology to determine how SNS relieves anxious insomnia.

To determine how SNS may protect against anxious insomnia, several genetic, molecular and network pharmacology strategies were used. These include protein-protein interaction (PPI) analysis, Gene Ontology (GO) and Kyoto Encyclopedia of Genes and Genomes (KEGG) enrichment analysis. We also applied molecular docking technology to further illustrate the mode of action of SNS at a cellular level and how this could be adopted for future animal experiments.

## 2 MATERIALS AND METHODS

### 2.1 Screening for Active Compounds of SNS and Prediction of Compound-related Target Genes

Four components in SNS (chaihu, baishao, zhishi and gancao) were respectively input into the Traditional Chinese Medicine System Pharmacology Database 2.3 (TCMSP database 2.3, https://tcmsp-e.com/tcmsp.php) and the database of the Traditional Chinese Medicines Integrated Database (TCMID, http://bidd.group/TCMID/). The active chemical compounds of these components were selected based on the following criteria: oral bioavailability (OB) ≥ 30%, drug-likeness (DL) ≥ 0.18, and drug half-life (HL) ≥ 4 hours. Among these standards, OB and HL are commonly used in network pharmacology as empirical criteria, while the DL value is calculated to ensure better alignment with ADME performance standards for comparison with drug compounds that have been systematically studied [24-26]. The TCMSP and TCMID databases are built on the systems pharmacology framework, specifically targeting the exploration of active compounds, target proteins, and related diseases in TCM. However, these databases may have some limitations in data download, lack sufficient quantifiable data, or face accessibility issues. In contrast to many other TCM-related databases (such as TCMdatabase@Taiwan, TCM-Mesh, and HIT), TCMSP and TCMID stand out by providing more extensive data and more convenient download options. Our selection criteria focus on whether they offer comprehensive ADME-related attributes such as oral bioavailability, half-life, drug-likeness, and blood-brain barrier permeability [27].

### 2.2 Collection of Anxious Insomnia-related Potential Target Genes

GeneCards database (https://www.genecards.org/) was used to search genes related to anxious insomnia by distributing both key words, such as “Generalized Anxiety Disorder” and “Insomnia” [28]. Therapeutic Target Database (TTD, http://db.idrblab.net/ttd/) and Comparative Toxicogenomic Database (CTD, http://ctdbase.org/) were used to supplement the targets of anxious insomnia [29-31]. The target genes induced by active compounds in SNS were cross-aligned with anxiety-related and insomnia-related genes to obtain the core target genes curing anxiety-induced insomnia. A Venn diagram was then prepared to understand the distribution of targets of the four herbs in order to provide some insight into the activity of SNS.

### 2.3 Construction and Topological Analysis of Components-Compounds-targets (CCT) Network

To understand the molecular mechanisms of SNS, a graph of the CCT network was constructed by Cytoscape 3.9.1 software (Cytoscape Team, USA) with topological parameters of nodes, including betweenness centrality (BC) and degree centrality (DC) [32, 33]. In the CCT network, DC ≥ 1.5 times median DC (DC ≥ 10) and BC ≥ 1 times median BC (BC ≥ 0.011) were used as the screening criteria to obtain the key compounds.

### 2.4 Protein-protein Interaction (PPI) Network Construction of Targets of SNS for Anxious Insomnia

To gain a comprehensive understanding of the molecular mechanisms, we used Cytoscape 3.9.1 software (Cytoscape Team, USA) and STRING database v11.5 (https://string-db.org/) to construct the PPI network [34, 35]. We selected PPI data with a combined score higher than 0.4 to ensure the network met both accuracy and quantity requirements. The combined score reflects confidence in the types of evidence for protein interactions, such as co-expression, phylogenetic co-occurrence, and gene fusion [36]. During the network construction process, we used the highest levels of betweenness centrality (BC), closeness centrality (CC), and degree centrality (DC) as criteria for identifying key compounds. Specifically, the screening criteria included degree centrality (DC) ≥ 1 times median DC (≥ 10), betweenness centrality (BC) ≥ 1 times median BC (≥ 0.011), and closeness centrality (CC) ≥ 1 times median CC (≥ 0.43). Additionally, proteins that did not interact with other proteins were excluded.

### 2.5 Function Enrichment Analysis of Gene Ontology (GO) and Kyoto Encyclopedia of Genes and Genomes (KEGG) Pathway

The 59 key protein targets of SNS for anxious insomnia were submitted to The Database for Annotation, Visualization, and Integrated Discovery (DAVID) bioinformatics resources (https://david.ncifcrf.gov/) in order to analyze GO function enrichment and KEGG pathway enrichment [37]. GO terms and KEGG pathways with a *p*-value less than 0.05 were considered statistically significant and valid [38]. Additionally, we identified representative pathways and GO terms for the target gene groups using the values of *p*-value (-log) and Fold Enrichment for ranking. This statistical analysis is more reliable compared to intuitive interpretations of data sets. However, there may still be slight discrepancies in the mechanistic validity of pathway analysis when compared with animal experiments involving multi-component drugs. Nonetheless, such research can serve as a preliminary data analysis before animal experiments, aiding in future experimental design.

### 2.6 Molecular Docking of Component-target Interaction of SNS against Anxious Insomnia

Molecular docking was analyzed using AutoDock 4.2 software, which employs the Lamarckian genetic algorithm [39]. All the three-dimensional structures of target proteins were downloaded from the RCSB PDB database (https://www.rcsb.org/), including AKT1 (pdb id: 1UNQ) [40], SLC6A4 (pdb id: 7LIA) [41], MAOA (pdb id: 6EZZ) [42], ACHE (pdb id: 2WG2) [43], CHRNA7 (pdb id: 3C84) [44], GABRA2 (pdb id: 5OSC) [45], ADRB2(pdb id: 2RH1) [46], ESR1 (pdb id: 7JKY) [47], CYP3A4 (pdb id: 5VCC) [48], HTR2A (pdb id: 7WC8) [49] and NOS3 (pdb id: 7TSM) [50]. In order to ensure the active conformation, we carefully selected target protein models from the RCSB PDB database obtained through X-ray crystallography and cryo-electron microscopy (Cryo-EM) techniques. Additionally, we considered only protein models with a resolution of less than 3.5Å to certify appropriate crystal quality [51]. For X-ray crystallography models, we employed an additional filtering criterion, requiring the R-Value Free to be below 0.25, further validating the accuracy of the selected models [52]. These pre-docking preparation parameters will be presented in Table **1**. In the process of preparing the target protein models, we first removed the original ligands from the PDB files, including H_2_O, free ions (such as Cu^2+^, Zn^2+^, Cl^-^, *etc.*), and the protein sequences. Next, we preserved the region corresponding to the orthostatic site of the original model and replaced it with our active compounds' “docking box.” All docking simulations were performed using default settings, with nine docking runs conducted to generate up to 9 different binding modes. We selected the docking result with the largest binding energy absolute value as the best receptor-ligand interaction simulation and presented its binding profile.

## 3 RESULTS

### 3.1 Potential Target Genes Analysis among Generalized Anxious Insomnia and SNS

For the anxious insomnia-related potential target genes analysis, a comparison of the potential target genes among generalized anxiety disorder, insomnia and SNS was screened using the GeneCards database, TTD, and CTD, TCMID database and TCMSP database. We used two different databases to provide target genes for SNS. The generalization principle of TCMSP involves extracting target genes separately by capturing four different components from SNS. On the other hand, TCMID extracts target genes from the perspective of the entire herbal formula. Five thousand five hundred seventy-four genes for generalized anxiety disorder, 2,906 genes for insomnia, 101 genes for SNS in TCMSP and 82 genes in TCMID were observed. Based on the Venn diagram analysis in Fig. (**1**), there were 1,438 overlapping potential target genes between generalized anxiety disorder and insomnia. These 1,438 genes related to generalized anxiety disorder and insomnia were further surveyed along with SNS potential target genes. The comparison of the potential target genes is shown in Fig. (**1**). The results reveal that there are 59 overlapping potential target genes among generalized anxiety disorder, insomnia, and SNS. Moreover, we retrospectively trace back these overlapped 59 potential target genes from the TCMSP and TCMID database, and then identified 31 compounds in SNS that can regulate these genes. Besides, there are a total of 779 compounds present in SNS. These compounds will be referred to as our active compounds. The active compounds in SNS and their corresponding potential target genes were summarized in Table **2**. Moreover, the corresponding structural formula of each active compound is listed in Fig. (**2**).

### 3.2 Network Construction and Topological Analysis

To understand the correlations between SNS and its four components with potential target genes related to anxious insomnia, a complex correlation network was constructed. As shown in Fig. (**3**), there were 96 nodes (consisting of 1 disease, SNS from TCMID database and its four components from TCMSP database, 31 active compounds and 59 potential target genes) and 278 edges. Results show that the size of orange squares and yellow circles reflects the statistical significance of these terms. Active compounds with the criterion of DC ≥ 10 and BC ≥ 0.011 were summarized. Table **3** shows that active compounds were selected according to node topological parameters. These active compounds in distinct components are β-sitosterol and kaempferol in baishao, stigmasterol and isorhamnetin in chaihu, shinpterocarpin in gancao and tetramethoxyluteolin, nobiletin and isosinensetin in zhishi.

### 3.3 Construction and analysis of the PPI network

We incorporated 59 target protein molecules (Table **S1**) and then conducted a PPI network analysis. As shown in Fig. (**4**), there were 59 nodes and 336 edges in the network. The results show that the three specific clusters in distinct colors (orange, green and yellow) were found to have a high functional correlation with neuron-related inflammatory responses and synaptic-neurotransmitter regulations. These include the following: the inflammatory response related cluster (including alpha serine/threonine-protein kinase (AKT1), interleukin-6 (IL-6), tumor necrosis factor (TNF), acetylcholinesterase (ACHE), estrogen receptor 1 (ESR1), cytochrome P450 3A4 (CYP3A4), cholinergic receptor Nicotinic Alpha 7 (CHRNA7), nitric Oxide Synthase 3 (NOS3), beta-2 adrenergic receptor (ADRB2), tumor protein 53 (TP53), solute carrier family 6 member 3 (SLC6A3) and peroxisome proliferator-activated receptor (PPAR)), the serotonergic/ dopaminergic synaptic related cluster (including sodium-dependent serotonin transporter (SLC6A4), 5-hydroxytryptamine receptor 2A (HTR2A), monoamine oxidase type A (MAOA), dopamine receptor D1(DRD1) and monoamine oxidase type B (MAOB)) and the gamma-aminobutyric acid synaptic related cluster (including gamma-aminobutyric acid receptor subunit alpha 2 (GABRA2), and muscarinic acetylcholine receptor 1 (CHRM1)). These findings suggest that SNS regulates anxious insomnia by ameliorating the synaptic system in the central nervous system and relieving the neuro-inflammatory response. After screening the network using the criteria of DC ≥10, BC ≥ 0.011, and closeness centrality ≥ 0.43, 19 proteins were selected and shown as the core target proteins in the PPI network analysis (Table **4**). Besides, from the width and transparency of the edges determined by the combined score, it is noticed that not only do the target proteins within the same protein cluster exhibit interaction tendencies, but there are also certain interaction tendencies between different protein clusters.

### 3.4 Functional and Pathway Enrichment Analysis

GO functional enrichment analysis was performed in Fig. (**5**) and Table **5**. The biological process annotations included chemical synaptic transmission, response to lipopolysaccharide, and positive regulation of the MAPK cascade. The cellular component annotations showed enrichment in neuron projection, synapses, neuronal cell bodies, integral components of the plasma membrane, and others. The molecular function annotation revealed neurotransmitter receptor activity, enzyme binding, protein heterodimerization activity, and protein homodimerization activity, among others.

A total of 84 pathways were identified in the KEGG signal pathway enrichment analysis. The Top 20 pathways were selected and listed in Fig. (**6**) and Table **6**. We see illustrated neuroactive ligand-receptor interaction pathways, pathways of neurodegeneration, *PI3K-AKT* signaling pathways and major types of synaptic pathways, such as GABAergic synapses, cholinergic synapses, serotonergic synapses, and dopaminergic synapses involved in emotion regulation.

### 3.5 Potential Target Spot Configuration of SNS in Synaptic Regulation Pathways

The neuroactive ligand-receptor interaction signaling pathway is the most critical aspect of the potential mechanisms of SNS treatment. Generally, most of the target genes were concentrated in serotonergic, cholinergic, dopaminergic, and GABAergic systems. The regulation of SNS, SSRIs (**e.g.*,* Fluoxetine) and benzodiazepines (**e.g.*,* Alprazolam) was evaluated see in Fig. (**7**). The genes involved in the regulation of SNS treatment mechanisms were more extensive compared to the treatment with Fluoxetine and Alprazolam. It appears that SNS, Fluoxetine, and Alprazolam are involved in the gene regulation of the serotonergic synapse pathway. By contrast, the cholinergic synapse pathway had only SNS and Fluoxetine involved. The GABAergic synapse pathway had only SNS and Alprazolam involved. It is noteworthy that SNS can target specific spots in the dopaminergic synapse system. In addition, significant targets of SNS treated anxious insomnia were distributed on the compressed “synaptic neurotransmitter regulation pathway”. The involved protein targets in SNS treatment of each synaptic system pathway are summarized in Table **7**.

### 3.6 The Construction of the Compounds-Targets- Synaptic Pathways (CTP) Network and Pre-docking Preparation

The CTP network was constructed by utilizing 20 associated targets listed in Table **7** and their corresponding 19 related compounds, which were identified through a reverse lookup in Table **2**. Also, the four synapse-associated pathways of SNS treatment in Fig. (**8**) were included. The construction of the CTP network was intended to narrow our research focus specifically to the scope of how SNS regulates the four synapse-associated pathways. We hope to gain a deeper understanding of the compounds that can effectively modulate diverse neural synaptic functions. Additionally, we observed that the core compounds selected from the CCT network analysis have a high degree of overlap with those in the CTP analysis. This reinforces the notion that the neural synaptic regulation mechanism is especially crucial in SNS treatment. Five active compounds (β-sitosterol, kaempferol, tetramethoxyluteolin, isorhamnetin, and shinpterocarpin) with high degree and BC values in the CCT network were also found in the CTP network, then were employed as molecular docking candidates of ligands. In this case, we excluded compounds C2, J2, and J5 from the lists of ligand candidates in Table **3**. Although J3, J2, and J5 are present in the CTP network, the regulatory interactions with target proteins are minimal (degree≤ 2). Therefore, we selected only J3, which has the higher degree and BC of the CCT network, as the representative compound for the component, zhishi. Additionally, while C2 demonstrated good parameters in both CCT and CTP networks, we decided not to include it as a ligand for molecular docking due to its high structural similarity to compound B4. β-sitosterol and stigmasterol share a high structural similarity, containing an ethyl group^37^ on their side chains compared to cholesterol. In contrast to β-sitosterol, stigmasterol has a double-bond in the side chain [53]. For receptor candidates, 11 target molecules (**e.g.*,* AKT1, SLC6A4, ADRB2, MAOA, ACHE, ESR1, CYP3A4, CHRNA7, GABRA2, HTR2A and NOS3) played important roles in both PPI network and the four synaptic system pathways were chosen by consulting with node topological parameters. Although the candidate pairs for molecular docking differ from the target-compound pairs shown in Table **2**, this molecular docking analysis will still be performed to validate the statistical results obtained from CCT, PPI, and CTP network analyses. It will also facilitate the execution of novel target-compound experiments with divergent results from the extant databases.

### 3.7 Molecular Docking Analysis of Compounds-targets Interaction

Five selected compounds (β-sitosterol and kaempferol in the baishao component, isorhamnetin in the chaihu component, shinpterocarpin in the gancao component and tetramethoxyluteolin in the zhishi component) were obtained after screening the CCT network and the CTP network. They were docked with AKT1, SLC6A4, ADRB2(Beta-2 adrenergic receptor), MAOA, ACHE (Acetylcholinesterase), ESR1 (Estrogen receptor 1), CYP3A4 (Cytochrome P450 3A4), CHRNA7 (Cholinergic Receptor Nicotinic Alpha 7), GABRA2, HTR2A (5-Hydroxytryptamine receptor 2A) and NOS3 (Nitric Oxide Synthase 3). Generally, the higher the binding energy absolute value, the greater the protein binding affinity of the ligand. As shown in Fig. (**9**), isorhamnetin-AKT1(9.9 kcal/mol), isorhamnetin-SLC6A4 (10.4 kcal/mol), β-sitosterol- MAOA (10.9 kcal/mol), β-sitosterol-ACHE (11.3 kcal/mol), isorhamnetin-CHRNA7 (10.3 kcal/mol) and shinpterocarpin-GABRA2 (11.1 kcal/mol), were markedly greater than 9.9 kcal/mol in absolute-value affinities and had more visualized polar hydrogen bond than other molecular docking pairs(at least one pair), indicated that the conformation of protein binding between these key compound-target pairs in the SNS treatment was stable. Therefore, the results in Fig. (**10**). showed that the active compound isorhamnetin forms hydrogen bonds with the amino acid residues of AKT1(TYR-38, ARG-48, ASN-54), CHRNA7(PRO-88) and SLC4A6(ARG-104, ASP-328, THR-497). β-sitosterol forms hydrogen bonds with the amino acid residues of MAOA(ALA-616) and ACHE(SER-286). Shinpterocarpin forms hydrogen bonds with the amino acid residues of GABRA2 (SER-28). These outcomes of molecular docking confirmed the results of the network pharmacology analysis regarding the anti-anxiety and anti-insomnia effects of SNS and established a springboard for further study of its pharmacological mechanisms.

## 4 DISCUSSION

In this study, we attempted to recognize all the statistical criteria of SNS components in the CCT network, KEGG analysis and CTP network. We found that the top five active compounds, β-sitosterol and kaempferol in baishao, isorhamnetin in chaihu, shinpterocarpin in gancao and tetramethoxyluteolin in zhishi, represented the most typical therapeutic compounds in SNS. Beta-sitosterol, a phytosterol found in baishao and commonly obtained through daily dietary ingestion, is acquired through intestinal absorption. Beta-sitosterol ingestion leads to the reduction of IL-6, TNF-α, and reactive oxygen species production, and it also suppresses the activation of the NF-κB signaling pathway [54]. Moreover, β-sitosterol could upregulate the resistance to lipid peroxidation and oxidative stress by means of estrogen receptor-mediated *PI3K/AKT/GSK-3β* signaling pathway in the dentate gyrus; this would have the function of relieving anxiety [55]. Kaempferol, a flavonoid from baishao with anti-inflammatory and anti-oxidative characteristics, inhibits PI3K and AKT phosphorylation, and decreases *TNF-α* and *IL-6* expression. Moreover, kaempferol increases GABA levels and decreases glutamine levels by activating glutamic acid decarboxylase and enhancing GABAergic synapse transmission in the hypothalamus [56, 57]. Isorhamnetin, one kind of O-methylated flavonol in chaihu, has the capability to protect neurons against oxidative damage, inflammation, and apoptosis by modifying *PI3K/AKT* and *NF-κB* signaling pathways. Isorhamnetin can enhance cholinergic signaling and synaptic plasticity by attenuating the activity of acetylcholinesterase and strengthening brain-derived neurotrophic factor expression in the prefrontal cortex as well as hippocampus [58]. Tetramethoxyluteolin, a derivative of flavonoid luteolin from Zhishi, is more metabolically stable and has greater oral absorption because of its methoxy groups. It can inhibit inflammatory reactions unique to the brain and has neuroprotective functions to process neurodegenerative diseases by diminishing gene expression and secretion of IL-6 and TNF-α. Tetramethoxyluteolin also acts as a potent inhibitor of the *PI3K/AKT/mTOR* signaling pathway [59]. Shinpterocarpin, a kind of isoflavone, is widely found in gancao and shows a high affinity for the GABA-BZD-chloride receptor, which results in considerable anxiolytic effects [60].

According to the analyses by the PPI network, the core target proteins of SNS treating anxious insomnia are AKT1, IL6, TNF, TP53, NOS3, and PPAR. The deficiency of activated AKT1 in laboratory animals resulted in functional and structural deviations in the prefrontal cortex and led to anxiety disorder or widespread cognitive behavioral deficits [61]. It has been observed that SNS exerts a dual modulatory effect on AKT phosphorylation (activation) through different mechanisms associated with diverse diseases. Experimental findings indicate both an enhancement and a suppression of AKT phosphorylation upon SNS treatment. Zhang. M. noted that SNS has demonstrated an elevation in AKT phosphorylation, which prevents excessive autophagy responses by activating the PI3K/AKT/mTOR pathways, mainly in hippocampal neurons, to counterbalance corticosterone-triggered neurotoxicity [62]. Conversely, paeoniflorin, a component found in baishao and also present in SNS, is reported to significantly diminish AKT phosphorylation, resulting in the inhibition of the NF-κB signaling route and subsequently curbing the production of inflammatory cytokines in cells [63, 64]. However, the current literature falls short in conclusively identifying whether SNS or its active ingredients specifically modulate which variant of serine/threonine-protein kinases, be it AKT1, AKT2, or AKT3. Therefore, upcoming studies should probe further into this matter to obtain a holistic grasp of SNS's influence on AKT and its role in the counteraction against anxiety-related insomnia. As IL-6 is elevated because of chronic stress, it causes expanded recruitment of proinflammatory monocytes and heightens the expression of their related gene, which is associated with the development of anxiety [65]. SNS exhibits a significant ability to reduce IL-6 levels in induced inflammatory tissues, demonstrating its role in anti-inflammatory and immune response modulation [66, 67]. TNF-α with correlative *JAK2-STAT3* signaling pathway plays a major role in the inflammatory response of neurons in the prefrontal cortex, which in turn is strongly linked to anxiety development [68]. Xiaoyaosan, a TCM-containing compound in SNS, exerts stress-induced anxiolytic-like effects by suppressing activation of the TNF-α induced *JAK2-STAT3* signaling pathway [69]. Moreover, IL-6 and TNF-α serve as presumptive factors in sleep [70]. Total secretion of IL-6 correlated inversely with both insomniac patients’ personally perceived quality of sleep and slow wave sleep and correlated positively with the awakening time in insomniac patients. These phenomena exhibited a vicious cycle between insomnia and inflammatory response [71]. The functions of these target proteins provide implicit evidence that SNS may remedy anxious insomnia by reducing inflammation, neuron degeneration, or cell autophagy.

Apart from anti-inflammatory-related mechanisms, neuroactive ligand-receptor interaction pathways, including various kinds of synaptic transmission pathways, provided vital clues and characteristics when screening SNS against anxious insomnia. In the PPI network, we picked up target proteins, like SLC6A4, ACHE, MAOA, CHRNA7 and GABRA2, in clusters more related to serotonergic, dopaminergic, and GABAergic synapse pathways as second-order core target proteins. These target proteins played crucial roles in synaptic functional management with the neurotransmitter mechanism of presynaptic promotion and reuptake diminishment. SLC6A4 promoter regions have functional polymorphism, which has been associated with manifold aspects of neuroticism and psychopathology, particularly anxiety disorder traits [72]. The up-expression of SLC6A4 in the pregenual cingulate cortex and amygdala may induce excessive arousal, hypothalamic-pituitary-adrenal axis system activation, or hypervigilance, which may frequently part of the process in anxious insomnia [73]. Increased serum acetylcholinesterase (ACHE), and accordingly decreased acetylcholine, can hence accentuate the release of pro-inflamed cytokines by macrophages related to insomnia. The neuroprotective function of inhibiting acetylcholinesterase activity and possessing antioxidant properties was accomplished by normalizing the AKT-mTOR signaling pathway, similar to how SSRIs ameliorated anxiety-like behaviors and hyperactivity [74]. Monoamine oxidase A (MAOA) has been associated with a large number of stress-induced psychological maladjustments because of the triggering of the HPA axis and the excitation of cortisol among the bilateral anterior hippocampus and other brain regions [75]. During post-translational modification, the promoter/exon/intron region DNA methylation in the *MAOA* gene can produce anxiety disorders [76]. *MAOA* expression also influences slumber by melatonin biosynthesis in ApoE4 cells. Rising melatonin levels in ApoE4 cells occur with down-regulation of MAOA expression, which affects metabolic enzymes through its precursor, serotonin, to cause insomnia [77]. GABA receptors α2, considered the chief inhibitory receptors for the central nervous system, can mediate anxiolytic-like activities, alleviate insomnia, and reduce hyperalgesic responses [78]. Besides, CHRNA7 regulates the release of both excitatory neurotransmitter glutamate and inhibitory neurotransmitter GABA in the hippocampus, cerebral cortex, and thalamus, which is associated with decreased hippocampal Glutamic acid decarboxylase and GABAα2 receptor levels [79]. Regrettably, animal experiments related to the therapeutic effects of SNS on neurotransmitters have not been widely conducted. Only a few network-pharmacology studies have confirmed the mechanism [80].

The KEGG enrichment analysis also demonstrated a similar result found in PPI network analysis, namely that the pathway terms could be separated into two classes: synaptic transmission-related terms and inflammatory response-related terms. In terms related to inflammatory response, the calcium signaling pathway, PI3K/AKT signaling pathway, cGMP-PKG signaling pathway, and estrogen signaling pathway were identified as potential mechanisms through which SNS may alleviate cell or neuron inflammation in anxious insomnia. The PI3K/AKT signaling pathway and calcium signaling pathway are crucial for neuronal survival and axonal regeneration following peripheral nerve injury. These pathways facilitate regeneration, neuron survival, and axon extension through BDNF downstream regulation [81]. Neuroactive ligand-receptor interaction and various synaptic pathways (serotonergic, cholinergic, dopaminergic, and GABAergic synapse pathways) are equivalently critical in SNS treatment mechanisms. In general, anxiety has been predominantly linked to expression reinforcement and the co-expression of catecholamines transporters in the hippocampus [82]. Studies have found that Suan-Zao-Ren Decoction, which includes gancao, improves sleep quality and enhances the binding affinity for serotonin receptors [83]. The appearance of GABA receptor allosteric sites results in amygdala neuron inhibitions, and these allosteric sites become molecular targets of anxiolytic drugs [84]. Studies have investigated the elevation of BDNF and GABA levels and have found that reducing cortical excitability ameliorates primary insomnia [85]. Glabrol from gancao inhibits flumazenil (a selective GABRA antagonist) by binding to GABAA-BZD receptors in rat cerebral cortical membranes [2]. These pathways largely overlap mechanisms of known anxiolytics and hypnotics, such as BZDs, SSRIs and SNS. We found that SNS has a wider range of regulatory sites and types, especially in serotonergic and GABAergic synapse pathways, and plays similar roles as SSRI and BZD. Furthermore, SNS also could regulate dopaminergic pathways, which were not involved in the mechanisms for SSRI and BZD. Therefore, we observed that the treatment mechanism of SNS is more extensive at the molecular level. Future research should investigate further comparisons with Western medicine and provide further clarification of pathway mechanisms for SNS.

The results of molecular docking demonstrated that greater protein binding affinities and more highly visualized polar hydrogen bonding displayed the greatest similarity to the actual structures. As shown in the affinity heat map, the docking pairs (isorhamnetin-AKT1, isorhamnetin-SLC6A4, β-sitosterol-MAOA, β-sitosterol-ACHE, isorhamnetin-CHRNA7, and shinpterocarpin-GABRA2) exhibited more stable conformations of ligand-receptor binding between key compounds and core target proteins in the SNS pathway. Isorhamnetin has been proposed to inhibit the phosphorylation cascade of AKT1 and its related signaling pathways, such as the PI3K/Akt/mTOR and MEK/ERK signaling pathways. Isorhamnetin acts as an AKT and MEK inhibitor, demonstrating anti-inflammatory and anti-autophagic effects. It effectively inhibits the proliferation of human breast cancer and gallbladder cancer cells, exhibiting a certain level of cytotoxicity inhibition [86, 87]. Additionally, β-sitosterol can alleviate neurotoxicity by inhibiting ACHE levels and has been shown to play an important role in the therapeutic mechanisms of many neurodegenerative diseases, such as Alzheimer's disease and dementia. In the study of Grewia optiva-related compounds molecular docking, it is suggested that β-sitosterol forms hydrogen bond interactions with the carbonyl group of Ser-286 in ACHE at the phenolic active site [88, 89]. These molecular modules corresponded with anti-inflammatory and neuroactive regulation as key tactics for SNS to treat anxious insomnia.

There are several limitations in our network-pharmacology and molecular docking analysis study. The data quality of TCM databases may be uncontrolled, incomplete, and heterogeneous, thus necessitating the establishment of uniform and rigorous standards. The application of network pharmacology in SNS research is mainly in the qualitative stage. Besides, there is a dose-response relationship between drugs and diseases. Our network pharmacology technology makes it difficult to achieve the goal of dose quantification. Further exploration is needed, particularly in new algorithm developments, designed animal experiments, and clinical applications, to gain a better understanding of the regulatory mechanisms of diseases and syndromes, as well as to elucidate the biological basis of SNS [90, 91]. In summary, at present, this is a computational result of empirical observation without rigorous animal or clinical trials, but it offers valuable perspectives for future in-depth research.

Our study, utilizing network pharmacology and molecular docking analysis, has several limitations. Unfortunately, the existing traditional Chinese medicine databases do not show that the components of SNS can regulate proteins related to anxiety insomnia outside the realm of neurotransmitters, nor do they provide effective evidence on how these components modulate these proteins (whether positive or negative regulation). We attempted to use the QIAGEN Ingenuity Pathway Analysis (IPA) software to simulate potential pathways involving proteins in the pathophysiological mechanisms, aiming to observe more practical regulatory conditions. However, our network pharmacology approach still faces challenges in quantifying this relationship in terms of dosage. This underscores the need for further advancements, particularly in the development of novel algorithms, meticulously designed animal studies, and clinical applications. Our further experimental project tends to confirm the anxiolytic effect of SNS extract on mice subjected to lipopolysaccharide-induced anxiety and p-chlorophenylalanine-induced serotonin depletion insomnia. Anxiety-like and insomnia-like behavior analysis was assessed using the elevated plus-maze test, light-dark box test, and open field test. Besides, to explore the potential mechanisms of SNS underlying the anti-inflammatory and neuroactive regulation, we will assess the expression levels of AKT1, MAOA, ACHE, SLC6A4, CHRNA7 and GABRA2 in the serum, hippocampus, medial prefrontal cortex, and amygdala by radioimmunoassay.

## CONCLUSION

We explored the potential compounds and protein targets of SNS for the treatment of anxious insomnia using network pharmacology and molecular docking strategies. The therapeutic mechanisms of SNS revolved around inhibiting nerve inflammation, regulating the performance of the neuroprotective effect, and modifying the transmission of neurons and neurotransmitters related to dopaminergic, serotonergic, GABAergic, and cholinergic system and their collaboration. Several core active compounds, including beta-sitosterol, kaempferol, tetramethoxyluteolin, isorhamnetin and shinpterocarpin and targets, *AKT1, IL6, TNF, SLC6A4, MAOA, ACHE, CHRNA7* and *GABRA2* were screened by the PPI network, the CCT network and the CTP network and proved to be important. The results showed that SNS plays a vital role in the treatment of sleep disorders associated with anxiety through “multi- compounds, multi-targets, and multi-pathways.” Follow up studies should investigate regulation of inflammatory processes, neuroactive ligand-receptor interactions, and kinds of neurotransmitters. The system analysis used in the present paper can provide a purely computational result and a theoretical basis for the treatment of anxious insomnia with SNS.

## Figures and Tables

**Fig. (1):**
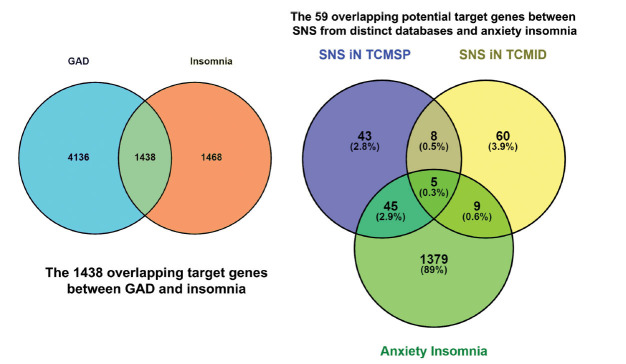
Potential target genes Venn screening among SNS from TCMSP database (entering four components separately), from TCMID database (entering SNS in the decoction form completely) and anxious insomnia.

**Fig. (2):**
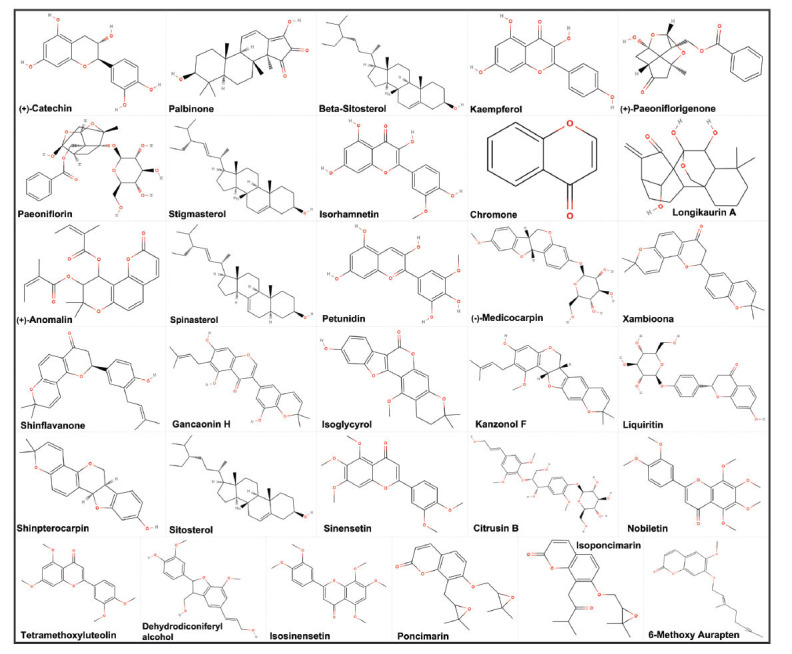
The corresponding structural formula of active compounds.

**Fig. (3):**
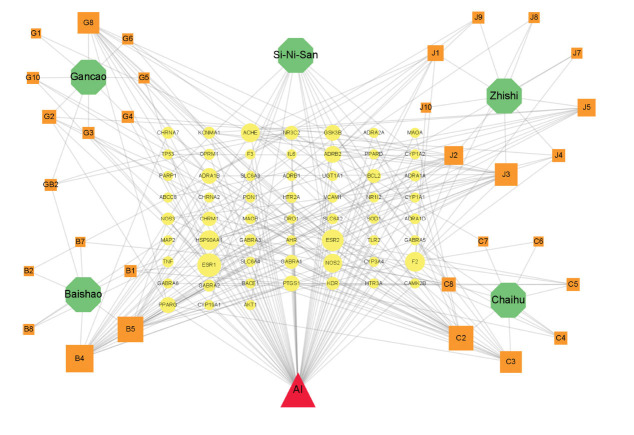
Components-compounds-targets networks of SNS and anxious insomnia. Red triangle is anxious insomnia. Green hexagons are SNS (from TCMID) and its four components (from TCMSP database). Orange squares indicated the active compounds of SNS components. Yellow circles indicated potential target genes. The size of nodes represents the DC and BC of target genes and compounds.

**Fig. (4):**
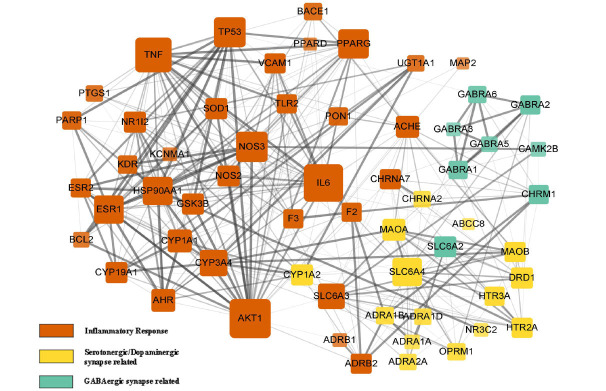
The interactive PPI network among 59 target proteins of SNS against anxious insomnia. There was a positive proportional relationship between the node size and the node degree criterion. The diverse width and transparency of the edges, which is determined by the combined score of two target proteins, represents the interaction extent of each node. Also, proteins with similar bio-functionality were classified into three distinct clusters.

**Fig. (5):**
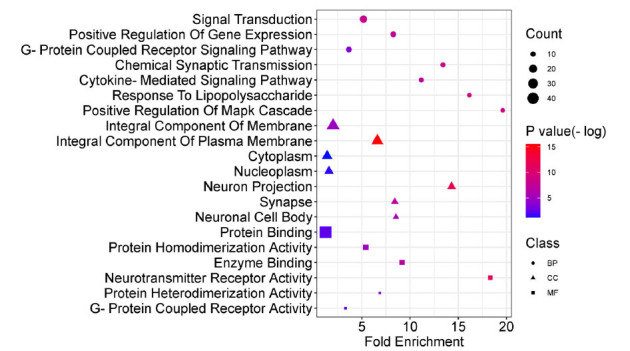
Functional distribution of GO enrichment annotation of target genes for SNS against anxious insomnia. The top 20 biological process (BP) terms, cellular component (CC) terms and molecular functions (MF) terms were exhibited according to the parameters (Gene counts ≥ 8 and *p*-value < 0.01). The distinct shapes showed the difference in GO terms. The y-axis was the gene functional classification of GO, and the x-axis was the corresponding value of fold enrichment. There was a positive proportional relationship between the size and the counts of the target genes. Moreover, a correlation exists between the color shade and the negative logarithm of the *p*-value, where darker red shades indicate higher values.

**Fig. (6):**
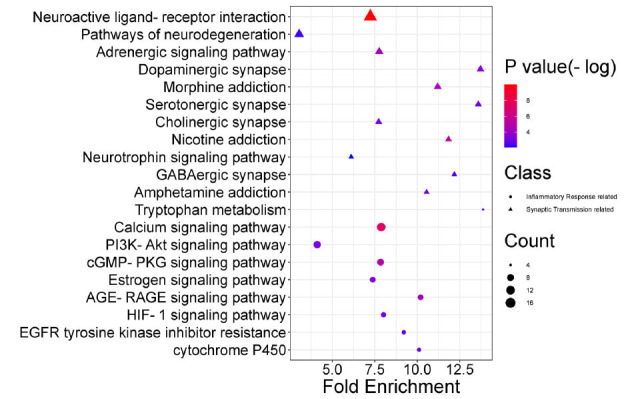
KEGG enrichment analysis of target genes for SNS against anxious insomnia. The color scales indicated the different thresholds of adjusted *p-*values, and the sizes of the dots represented the gene count of each term. The y-axis was the classification of the KEGG pathway, and the x-axis was the level of fold enrichment. Also, the distinct shapes showed the related functional difference of each pathway (synaptic transmission related and inflammatory response related).

**Fig. (7):**
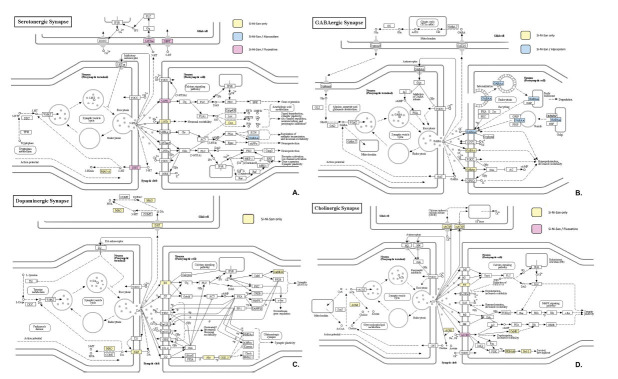
Potential target spots of SNS / SSRIs (Fluoxetine)/ benzodiazepines (Alprazolam) by regulating serotonergic synapse (**A**), GABAergic synapse (**B**), dopaminergic synapse (**C**), and cholinergic synapse pathways (**D**). Arrows indicate activation effects, T-arrows indicate inhibition effects and segments indicate activation effects or inhibition effects. The spots in yellow are targets regulated separately only by SNS; the red spots indicate regulation by SNS and Fluoxetine, and the blue spots indicate regulation by SNS and Alprazolam.

**Fig. (8):**
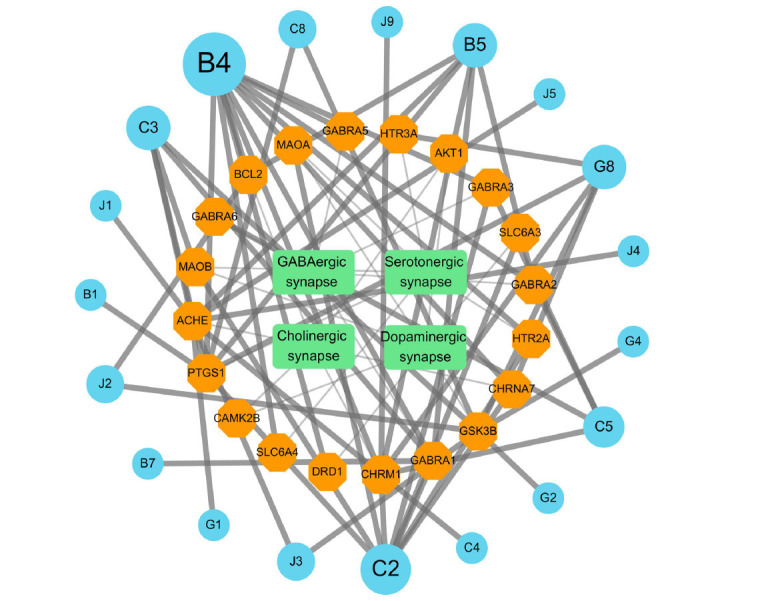
The compounds-targets-synaptic pathways network of SNS. Green squares indicated types of synaptic pathways. Orange circles indicated target genes. Blue hexagons indicate the active components of SNS. The degree values of the compounds are reflected by the size of the blue hexagons.

**Fig. (9):**
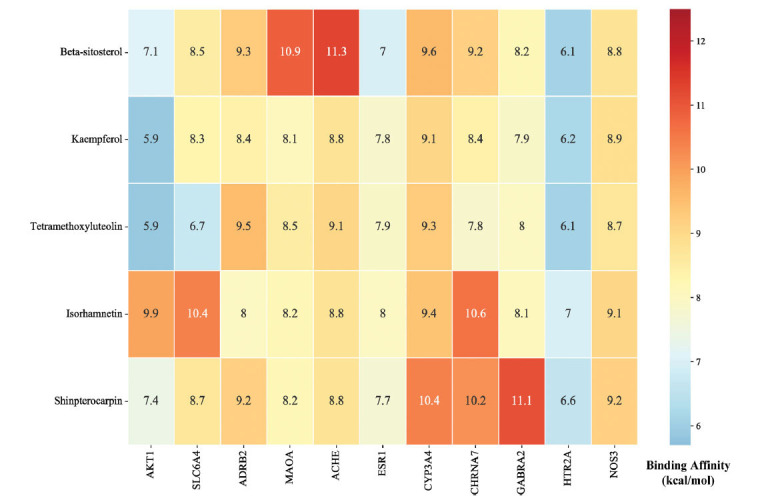
The heat map information of the compounds-targets binding affinity (showed in absolute value).

**Fig. (10):**
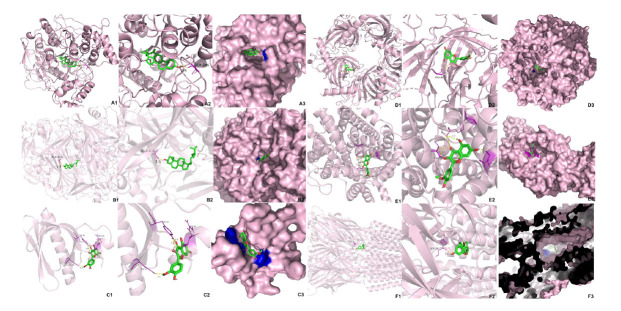
3D molecular docking visualization results of target proteins with active compounds of SNS. (**A**) β-sitosterol with ACHE (2WG2); (**B**) β-sitosterol with MAOA (6EZZ); (**C**) isorhamnetin with AKT1 (1UNQ); (**D**) isorhamnetin with CHRNA7 (3C84); (**E**) isorhamnetin with SLC6A4 (7LIA); (**F**) shinpterocarpin with GABRA2 (5OSC). Active compounds were represented by green-red ball-and-stick models, and the secondary structure of the protein was represented by a light pink ribbon. The yellow dotted lines between active compounds and target proteins represent the polar hydrogen bond bonding sides. In addition, surface conservation analysis emphasized how receptor compounds bind to ligand protein.

**Table 1:** The pre-docking preparation parameters of target proteins.

**SAMPLE**	**PDB ID**	**Method**	**Resolution**	**R-Value Free**
AKT1	1UNQ	X-ray Diffraction	0.98 Å	0.179
SLC6A4	7LIA	Electron Microscopy	3.3 Å	Nil
ADRB2	6EZZ	X-ray Diffraction	1.8 Å	0.197
MAOA	2WG2	X-ray Diffraction	1.95 Å	0.196
ACHE	3C84	X-ray Diffraction	1.94 Å	0.215
ESR1	5OSC	X-ray Diffraction	3.10 Å	0.246
CYP3A4	2RH1	X-ray Diffraction	2.4 Å	0.232
CHRNA7	7JKY	X-ray Diffraction	1.16 Å	0.176
GABRA2	5VCC	X-ray Diffraction	1.7 Å	0.204
HTR2A	7WC8	X-ray Diffraction	2.45 Å	0.244
NOS3	7TSM	X-ray Diffraction	1.85 Å	0.237

**Table 2:** The active compounds of SNS and their corresponding potential target gene.

**Molecule Name**	**Scientific Name**	**No.**	**Related Target Gene**
(+)-Catechin	*Paeonia lactiflora*	B1	PTGS1, ESR1, HSP90AA1
Palbinone	*Paeonia lactiflora*	B2	NR3C2
Beta-sitosterol	*Paeonia lactiflora*	B4	PTGS1, HSP90AA1, DRD1, CHRM1, GABRA2, HTR2A, GABRA5, ADRA1A, GABRA3, ADRA1B, ADRB2, CHRNA2, SLC6A4, OPRM1, GABRA1, CHRNA7, BCL2, PON1, MAP2
Kaempferol	*Paeonia lactiflora*	B5	NOS2, PTGS1, PPARG, HSP90AA1, F2, CHRM1, NOS3, GABRA2, ACHE, SLC6A2, ADRA1B, GABRA1, BCL2, TNF, CYP3A4, VCAM1, AHR
(+)-Paeoniflorigenone	*Paeonia lactiflora*	B7	GABRA1
Paeoniflorin	*Paeonia lactiflora*	B8	TNF, IL6
Stigmasterol	*Bupleurum chinense DC.*	C2	NR3C2, PTGS1, ADRA2A, SLC6A2, SLC6A3, ADRB2, MAOB, MAOA, CHRM1, ADRB1, HTR2A, ADRA1A, GABRA3, ADRA1B, GABRA1, CHRNA7
Isorhamnetin	*Bupleurum chinense DC.*	C3	NOS2, PTGS1, ESR1, PPARG, ESR2, GSK3B, HSP90AA1, PPARD, F2, NOS3, ACHE, GABRA1, MAOB
Chromone	*Bupleurum chinense DC.*	C4	F2, ESR1, ACHE, ESR2
Longikaurin A	*Bupleurum chinense DC.*	C5	CHRM1, GABRA2, GABRA3, GABRA6
(+)-Anomalin	*Bupleurum chinense DC.*	C6	F2
Spinasterol	*Bupleurum chinense DC.*	C7	NR3C2
Petunidin	*Bupleurum chinense DC.*	C8	NOS2, PTGS1, ESR2, GSK3B, HSP90AA1
(-)-Medicocarpin	*Glycyrrhiza uralensis*	G1	ACHE
Xambioona	*Glycyrrhiza uralensis*	G10	ESR1, ESR2, NOS2
Shinflavanone	*Glycyrrhiza uralensis*	G2	ESR1, ESR2, GSK3B, NOS2, PPARG
Gancaonin H	*Glycyrrhiza uralensis*	G3	ESR1, HSP90AA1, KDR
Isoglycyrol	*Glycyrrhiza uralensis*	G4	ESR1, GSK3B, NOS2
Kanzonol F	*Glycyrrhiza uralensis*	G5	ESR1, ESR2
Liquiritin	*Glycyrrhiza uralensis*	G6	KDR
Shinpterocarpin	*Glycyrrhiza uralensis*	G8	ADRA1B, ADRA1D, ADRB2, CHRM1, CHRNA7, ESR1, ESR2, GSK3B, HTR3A, NOS2, OPRM1, PPARG, PTGS1
Sitosterol	*Paeonia lactiflora /Glycyrrhiza uralensis*	GB2	NR3C2
Sinensetin	*Citrus aurantium*	J1	NOS2, F2, ACHE, ADRB2, ESR2, HSP90AA1, PTGS1, ADRA1B
Citrusin B	*Citrus aurantium*	J10	F2
Nobiletin	*Citrus aurantium*	J2	NOS2, PTGS1, F2, ESR1, PPARG, ESR2, HSP90AA1, KCNMA1, GSK3B, BCL2, TP53
Tetramethoxyluteolin	*Citrus aurantium*	J3	NOS2, PTGS1, F2, PPARG, NOS3, ADRA1B, ADRB2, ESR2, BACE1, GSK3B, HSP90AA1, ACHE, ADRA1D, NR1I2, KCNMA1
Dehydrodiconiferyl alcohol	*Citrus aurantium*	J4	F2, ESR1, HSP90AA1, ACHE
Isosinensetin	*Citrus aurantium*	J5	ESR1, PPARG, NOS3, ACHE, ADRB2, ESR2, HSP90AA1, KCNMA1
Poncimarin	*Citrus aurantium*	J7	F2, ESR1
Isoponcimarin	*Citrus aurantium*	J8	F2, ADRB2
6-Methoxy Aurapten	*Citrus aurantium*	J9	CHRM1, ADRA1B, ADRB2

**Table 3:** Topological analysis of the core compounds in components-compounds-targets network.

**No.**	**Compound Name**	**Betweenness Centrality**	**Degree**
B4	Beta-sitosterol	0.0557	20
B5	Kaempferol	0.0611	18
C2	Stigmasterol	0.0431	17
J3	Tetramethoxyluteolin	0.0316	15
G8	Shinpterocarpin	0.0359	14
C3	Isorhamnetin	0.0351	14
J2	Nobiletin	0.0182	11
J5	Isosinensetin	0.0143	10

**Table 4:** Topological analysis of the core target proteins in PPI network.

**Target Name**	**Degree**	**Betweenness Centrality**	**Closeness Centrality**
AKT1	32	0.1684	0.674
IL6	30	0.0835	0.659
TNF	27	0.0397	0.585
TP53	22	0.052	0.568
NOS3	22	0.0508	0.552
PPARG	21	0.0426	0.552
SLC6A4	20	0.1026	0.585
ESR1	20	0.0514	0.574
CYP3A4	18	0.0514	0.557
SLC6A3	17	0.073	0.574
ACHE	13	0.0364	0.547
MAOA	13	0.0197	0.495
DRD1	12	0.0246	0.449
CHRNA7	11	0.0339	0.537
ADRB2	11	0.03	0.537
MAOB	11	0.0138	0.483
HTR2A	11	0.0228	0.475
CHRM1	10	0.0196	0.456
GABRA2	10	0.0237	0.432

**Table 5:** Topological analysis of the GO functional enrichment analysis.

**Class**	**Term**	**Fold Enrichment**	***p* value(-log)**	**Count**
BP	Signal Transduction	5.17	8.14	19
BP	Positive Regulation Of Gene Expression	8.26	7.47	13
BP	G-Protein Coupled Receptor Signaling Pathway	3.65	3.83	13
BP	Chemical Synaptic Transmission	13.41	8.17	11
BP	Cytokine-Mediated Signaling Pathway	11.16	7.42	11
BP	Response To Lipopolysaccharide	16.15	9.08	10
BP	Positive Regulation Of Mapk Cascade	19.62	8.98	10
CC	Integral Component Of Membrane	2.02	4.82	32
CC	Integral Component Of Plasma Membrane	6.61	15.55	28
CC	Cytoplasm	1.4	1.16	22
CC	Nucleoplasm	1.59	1.4	18
CC	Neuron Projection	14.31	11.92	15
CC	Synapse	8.42	6.92	12
CC	Neuronal Cell Body	8.54	5.7	10
MF	Protein Binding	1.24	2.29	49
MF	Protein Homodimerization Activity	5.41	5.05	12
MF	Enzyme Binding	9.16	6.62	11
MF	Neurotransmitter Receptor Activity	18.32	11.43	10
MF	Protein Heterodimerization Activity	6.87	3.85	8
MF	G-Protein Coupled Receptor Activity	3.3	2.02	8

**Table 6:** Topological analysis of top 20 pathways in KEGG enrichment analysis.

**Class**	**Term**	**Fold Enrichment**	***p* value**	**Count**	**Genes**
Synaptic Transmission related	Neuroactive ligand-receptor interaction	7.2	10	18	GABRA2, GABRA1, CHRM1, CHRNA2, GABRA6, GABRA5, CHRNA7, GABRA3, ADRA1D, ADRB1, ADRB2, HTR2A, OPRM1, F2, ADRA1B, ADRA1A, ADRA2A, DRD1
Synaptic Transmission related	Pathways of neurodegeneration	3.1	2.4	10	CAMK2B, GSK3B, IL6, CHRM1, NOS2, CHRNA7, BCL2, TNF, SLC6A3, SOD1
Synaptic Transmission related	Adrenergic signaling pathway	7.8	4.2	8	CAMK2B, BCL2, AKT1, ADRA1D, ADRB1, ADRB2, ADRA1B, ADRA1A
Synaptic Transmission related	Dopaminergic synapse	13.7	3.6	7	CAMK2B, GSK3B, MAOB, MAOA, AKT1, DRD1, SLC6A3
Synaptic Transmission related	Morphine addiction	11.2	4.5	7	GABRA2, GABRA1, GABRA6, GABRA5, GABRA3, DRD1, OPRM1
Synaptic Transmission related	Serotonergic synapse	13.6	3	6	MAOB, MAOA, HTR3A, HTR2A, SLC6A4, PTGS1
Synaptic Transmission related	Cholinergic synapse	7.7	3	6	CAMK2B, ACHE, CHRM1, CHRNA7, BCL2, AKT1
Synaptic Transmission related	Nicotine addiction	11.8	5.2	6	GABRA2, GABRA1, GABRA6, GABRA5, CHRNA7, GABRA3
Synaptic Transmission related	Neurotrophin signaling pathway	6.1	2.1	5	CAMK2B, GSK3B, BCL2, AKT1, TP53
Synaptic Transmission related	GABAergic synapse	12.2	2.5	5	GABRA2, GABRA1, GABRA6, GABRA5, GABRA3
Synaptic Transmission related	Amphetamine addiction	10.6	2.9	5	CAMK2B, MAOB, MAOA, DRD1, SLC6A3
Synaptic Transmission related	Tryptophan metabolism	13.9	2.6	4	MAOB, MAOA, CYP1A2, CYP1A1
Inflammatory Response related	Calcium signaling pathway	7.9	7.3	13	CAMK2B, CHRM1, NOS2, NOS3, CHRNA7, ADRA1D, ADRB1, ADRB2, HTR2A, ADRA1B, ADRA1A, KDR, DRD1
Inflammatory Response related	PI3K-Akt signaling pathway	4.1	3.3	10	GSK3B, IL6, HSP90AA1, CHRM1, NOS3, BCL2, KDR, AKT1, TP53, TLR2
Inflammatory Response related	cGMP-PKG signaling pathway	7.8	4.9	9	NOS3, KCNMA1, AKT1, ADRA1D, ADRB1, ADRB2, ADRA1B, ADRA1A, ADRA2A
Inflammatory Response related	Estrogen signaling pathway	7.4	3.5	7	HSP90AA1, NOS3, BCL2, AKT1, OPRM1, ESR1, ESR2
Inflammatory Response related	AGE-RAGE signaling pathway	10.2	4.3	7	IL6, VCAM1, NOS3, BCL2, AKT1, TNF, F3
Inflammatory Response related	HIF-1 signaling pathway	8	3.1	6	CAMK2B, IL6, NOS2, NOS3, BCL2, AKT1
Inflammatory Response related	EGFR tyrosine kinase inhibitor resistance	9.2	2.7	5	GSK3B, IL6, BCL2, KDR, AKT1
Inflammatory Response related	cytochrome P450	10.1	2.9	5	MAOB, UGT1A1, MAOA, CYP1A2, CYP3A4

**Table 7:** The involved protein targets the SNS of each synaptic system.

**Pathways**	**Target Genes**
Cholinergic synapse	AKT1, BCL2, ACHE, CAMK2B, CHRM1, CHRNA7
Serotonergic synapse	HTR2A, HTR3A, MAOA, MAOB, PTGS1, SLC6A4
Dopaminergic synapse	AKT1, CAMK2B, DRD1, GSK3B, MAOA, MAOB, SLC6A3
GABAergic synapse	GABRA1, GABRA2, GABRA3, GABRA5, GABRA6

## Data Availability

The data used to support the findings of this study are included within the article.

## LIST OF ABBREVIATIONS

HTR2A: 5-Hydroxytryptamine Receptor 2A
ADRB2: Beta-2 Adrenergic Receptor
AKT1: Alpha Serine/Threonine-protein Kinase 1
BZD: Benzodiazepine
Biological: Betweenness Centrality
BP: Biological Process
CC: Cellular Component
CTD: Comparative Toxicogenomic Database
CHRNA7: Cholinergic Receptor Nicotinic Alpha 7
CCT: Components-Compounds-Targets
CTP: Compounds-Targets-Synaptic Pathways
CYP3A4: Cytochrome P450 3A4
DC: Degree Centrality
DRD1: Dopamine Receptor D1
ESR1: Estrogen Receptor 1
GABRA2: Gamma-aminobutyric Acid Receptor Subunit Alpha 2
GO: Gene Ontology
IL-6: Interleukin-6
KEGG: Kyoto Encyclopedia of Genes and Genomes
MF: Molecular Functions
MAOA: Monoamine Oxidase Type A
MAOB: Monoamine Oxidase Type B
CHRM1: Muscarinic Acetylcholine Receptor 1
NOS3: Nitric Oxide Synthase 3
PPAR: Peroxisome Proliferator-activated Receptor
PPI: Protein-protein Interaction
SSRI: Selective Serotonin Reuptake Inhibitors
SNS: Si-Ni-San
SLC6A4: Sodium-dependent Serotonin Transporter
SLC6A3: Solute Carrier Family 6 Member 3
DAVID: The Database for Annotation Visualization and Integrated Discovery
TTD: Therapeutic Target Database
TCM: Traditional Chinese Medicine
TCMSP: Traditional Chinese Medicine System Pharmacology
TCMID: Traditional Chinese Medicines Integrated Database
TNF: Tumor Necrosis Factor
TP53: Tumor Protein 53
