# Supplementary material for: Multi-target Mechanisms of Si-Ni-San on Anxious Insomnia: An Example of Network-pharmacology and Molecular Docking Analysis
Source: Curr Med Chem. 2024 Oct 9;32(13):2640–63. doi: 10.2174/0109298673299665240924090617 (PMC12307960; doi:10.2174/0109298673299665240924090617)
Supplement: Supplementary file 1 [file CMC-32-13-2640_SD1.pdf]

Supplementary Material

Multi-target Mechanisms of Si-Ni-San on Anxious Insomnia: An Example of Network-pharmacology and Molecular Docking Analysis

Chih Ting Lin[1,2,*], Hsin Yi Lin[3], Wen Huang Peng[3] and Lung Yuan Wu[1,4,5,*]

[1]The School of Chinese Medicine for Post-Baccalaureate, I-Shou University, No. 8, Yida Rd., Jiaosu Village Yanchao District, Kaohsiung City, 82445, Taiwan; [2]Department of Chinese Medicine, E Da Cancer Hospital, I Shou University, No. 21, Yida Rd., Jiaosu Village Yanchao District, Kaohsiung City, 82445, Taiwan; [3]School of Chinese Pharmaceutical Sciences and Chinese Medicine Resources, China Medical University, No.91, Hsueh-Shih Road, Taichung, 40402, Taiwan; [4]Wu Lung-Yuan Chinese Medicine Clinic, 3 F, No. 131, Section 1, Roosevelt Rd., Zhongzheng District, Taipei City, 10093, Taiwan; [5]Graduate Institute of Chinese Pharmaceutical Sciences, College of Chinese Medicine, China Medical University, No.91, Hsueh-Shih Road, Taichung, 40421, Taiwan

Table S1.

Gene Symbol	Description
ESR1	Estrogen receptor
HTR3A	5-hydroxytryptamine receptor 3A
OPRM1	Mu-type opioid receptor
SOD1	Superoxide dismutase
ACHE	Acetylcholinesterase
NR3C2	Mineralocorticoid receptor
CHRNA7	Neuronal acetylcholine receptor subunit alpha-7
GSK3B	Glycogen synthase kinase-3 beta
PPARG	Peroxisome proliferator-activated receptor gamma
ESR2	Estrogen receptor beta
ADRB2	Beta-2 adrenergic receptor
NOS2	Nitric oxide synthase
HSP90AA1	Heat shock protein HSP 90-alpha
CHRM1	Muscarinic acetylcholine receptor M1
KDR	Vascular endothelial growth factor receptor 2
PTGS1	Prostaglandin G/H synthase 1
ADRA1D	Alpha-1D adrenergic receptor
ADRA1B	Alpha-1B adrenergic receptor
TP53	Cellular tumor antigen p53
F2	Prothrombin
NOS3	Nitric oxide synthase
ABCC8	ATP-binding cassette sub-family C member 8
KCNMA1	Calcium-activated potassium channel subunit alpha-1
BCL2	Apoptosis regulator Bcl-2

BACE1	Beta-secretase 1
NR1I2	Nuclear receptor subfamily 1 group I member 2
SLC6A4	Sodium-dependent serotonin transporter
HTR2A	5-hydroxytryptamine receptor 2A
TNF	Tumor necrosis factor
IL6	Interleukin-6
SLC6A2	Sodium-dependent noradrenaline transporter
GABRA1	Gamma-aminobutyric acid receptor subunit alpha-1
GABRA2	Gamma-aminobutyric acid receptor subunit alpha-2
CYP3A4	Cytochrome P450 3A4
PON1	Serum paraoxonase/arylesterase 1
DRD1	Dopamine receptor 1A
GABRA5	Gamma-aminobutyric acid receptor subunit alpha-5
CHRNA2	Neuronal acetylcholine receptor subunit alpha-2
GABRA3	Gamma-aminobutyric acid receptor subunit alpha-3
MAP2	Microtubule-associated protein 2
ADRA1A	Alpha-1A adrenergic receptor
VCAM1	Vascular cell adhesion protein 1
AHR	Aryl hydrocarbon receptor
SLC6A3	Sodium-dependent dopamine transporter
MAOA	Amine oxidase
MAOB	Amine oxidase
ADRA2A	Alpha-2A adrenergic receptor
GABRA6	Gamma-aminobutyric acid receptor subunit alpha-6
ADRB1	Beta-1 adrenergic receptor
PPARD	Peroxisome proliferative activated receptor
CYP1A2	Cytochrome P450 1A2
AKT1	RAC-alpha serine/threonine-protein kinase
CYP19A1	Aromatase
F3	Tissue factor
UGT1A1	UDP-glucuronosyltransferase 1A1
TLR2	Toll-like receptor
CAMK2B	Calcium/calmodulin-dependent protein<br>-kinase type II subunit beta
CYP1A1	Cytochrome P450 1A
PARP1	ADP-ribose polymerase 1
